# Identifying the difference in time perception between major depressive disorder and bipolar depression through a temporal bisection task

**DOI:** 10.1371/journal.pone.0277076

**Published:** 2022-12-05

**Authors:** Panqi Liu, Hua Guo, Ruihua Ma, Sijia Liu, Xuan Wang, Ke Zhao, Yunlong Tan, Shuping Tan, Fude Yang, Zhiren Wang

**Affiliations:** 1 Peking University Huilonguan Clinical Medical School, Beijing Huilongguan Hospital, Beijing, China; 2 Zhumadian Mental Hospital, Zhumadian, Henan Province, China; 3 State Key Laboratory of Brain and Cognitive Science, University of the Chinese Academy of Sciences, Beijing, China; State University of New York Upstate Medical University, UNITED STATES

## Abstract

**Background:**

It is difficult to make a precise diagnosis to distinguish patients with Major Depressive Disorder (MDD) from patients with Bipolar Depressive Disorder (current depressive episode, BD). This study will explore the difference in time perception between MDD and BD using a temporal bisection task.

**Methods:**

In this temporal bisection task, 30 MDD patients, 30 BD patients, and 30 healthy controls (HC) had to categorize a signal duration, between 400 and 1600 milliseconds (ms), as either short or long. A repeated measurement analysis of variance with 3 (subject type) × 7 (time interval) was performed on the long response ratio with Bonferroni correction for multiple comparisons. Origin software was used to calculate the subjective bisection point (BP), difference limen (DL), and Weber ratio (WR). The Hamilton Depression Rating Scale for depression-17 was used to assess depressive symptoms in the patients.

**Results:**

The data showed that the interaction effect between subject type and duration was significant (*F*_(6,498)_ = 4.656, *p* <0.001, η^2^_*p*_ = 0.101). At 400 ms, and the long response of the MDD group was greater than HC group (*p*<0.017, Bonferroni-corrected). At 1200, 1400 and 1600 ms, the long response of BD group is smaller than HC group, (*p*<0.017, Bonferroni-corrected). The one-way ANOVA revealed significant difference among the HC, MDD and BD groups in the BP values WR values, *F*_(2, 81)_ = 3.462, *p* = 0.036 vs. *F*_(2, 81)_ = 3.311, *p* = 0.042. Post-hoc tests showed that the value of BP in the MDD group was less than BD group (*p* = 0.027) and the value of BP in the MDD group was less than HC group (*p* = 0.027), while there was not significant difference of BP values between BD group and HC group. The WR values in MDD group larger than the HC group (*p* = 0.022).

**Limitations:**

Severity of depression not divided and analyzed according to the Hamilton Depression Rating Scale score.

**Conclusion:**

The time perception of the MDD and BD groups was different from that of the HC group, they overestimated short time periods. Compared with the BD group, the MDD group had a smaller time bisector, and these patients felt that time passed more slowly. The time sensitivity of MDD group and BD group were less than the HC group. However, there was no statistical difference in time sensitivity between the MDD and BD groups.

## Introduction

Major depressive disorder (MDD; also referred to as depression) and bipolar disorder (previously referred to as manic-depressive disorder) are two common psychiatric disorders, collectively referred to as mood disorders or affective disorders.

MDD is a group of mood or affective disorders caused by depressive symptoms. It is the most common mental disorder with a lifetime prevalence of 16%, affecting one in five women and one in eight men [1]. According to the World Health Organization, by 2030, MDD will surpass cardiovascular disease to become the most debilitating disorder in the world [2]. Typical depressive episodes have three “low" symptoms of depression, which are at least two weeks with low mood, low energy, and low interest in normally enjoyable activities. In addition to emotional symptoms, patients with depression also have varying degrees of cognitive decline. This results in varying degrees of social dysfunction, causes life problems to the patient, and places a heavy burden on the family and on society [3].

Bipolar disorder, although not as prevalent as MDD, is considered to be one of the most burdensome psychotic diseases, affecting 1.5% to 3.0% of the world’s population [4, 5]. Bipolar disorder is characterized by recurrent periods of manic and depressive symptoms, and a mixed state during which there is a period of relatively stable mood. Patients with bipolar disorder invariably have varying degrees of neurocognitive impairment and have a high degree of suicidal tendencies. According to the Fifth Edition of the Diagnostic and Statistical Manual of Mental Disorders (DSM-V), bipolar disorder has two main types: Bipolar I Disorder (BD I) and Bipolar II Disorder (BD II). bipolar disorder, in the manic episode, is easy to diagnose for typical symptoms. However, as bipolar disorder consistently shows clinical symptoms similar to those of MDD in the period of depression (bipolar depression, BD), it is difficult to accurately distinguish the two diseases. Some studies have found that patients with BD and depression are 10 times more likely to be manic, and about 60% of BD patients with depression have been misdiagnosed with MDD, with an average period of misdiagnosis lasting 7.5–9.8 years [6, 7]. Inappropriate treatment of patients with misdiagnosed BD increases the risk of manic episodes, the likelihood of switching to a rapid cyclothymic disorder, and the rate of suicide rate in patients. It can take 10 years or longer to administer the correct diagnosis for those with BD during the period of depression, which means that misdiagnosed patients will spend more in medical costs, will impose a heavy burden on their family and society for a longer time, and will suffer greater pain overall. The current diagnosis of the two disorders is mainly based on clinical features, such as medical history, clinical symptoms, and course of disease [8, 9]. Currently, no objective, clinical markers are available. Therefore, there is a pressing need in psychiatric studies to find objective, biological diagnostic markers, explore the pathogenesis of MDD and BD, and provide evidence and guidance for its identification, clinical intervention and treatment.

Time perception is one of the most basic cognitive processes in humans. The ability to accurately perceive time plays an important role in daily life, impacting everything from the sleep–wake cycle to the ability to function throughout the day. Time is essential for activities, especially when making decisions and evaluating the environment [10–12]. Some studies have found that the subjective experience of time does not necessarily reflect the objective passing of time, because time perception is often influenced by a larger number of factors such as emotion, attention, anxiety, and memory [13–15]. In fact, the experience of emotion may have the strongest influence on time perception [16, 17]. When people feel happy or are in a good mood, they say that time flies; yet when they are bored or unhappy, they say the opposite.

Many studies show that when compared with healthy people, MDD patients have different experiences in terms of time. When MDD patients are in a depressed state, they feel that time passes slowly and describe their experiences in terms like “Time seems to be extended,” and “Every day is so long” [14, 18–20]. Some studies have suggested that time perception is affected by depression. For example, a time reproduction task used in Mahlberg R’s studies [21] showed that when 30 patients with acute depression participated in the task, they over-reproduced the short time interval. Wyrick RA [22] and Bschor et al. [14] showed that depression patients overestimated time intervals of 7, 35, and 90 seconds. A meta-analytic study [23] found that patients with depression tended to perceive time to pass more slowly, and compared to healthy control, depressive patients showed a tendency towards overproduction of short and underproduction of long time interval. However, Droit-Volet‘s study showed that moderately and severely depressed people underestimated the time interval, and that the higher the depression score, the larger the value of the bisection point (BP) from a temporal bisection task [24]. Limited data exist on time perception in bipolar disorder patients with both, manic and depression. BD patients, like MDD patients, often perceive time as going by very slowly [25]. In extreme situations, they even think time has stopped [19, 26]. However, when bipolar disorder patients are in the manic period, patients have different experiences. Previous studies have shown that manic bipolar disorder patients feel that time passes quickly and accelerates abnormally [19, 27–29]. A time discrimination task was used in Bschor’s study, which found that manic patients overestimated time intervals [14]. Evidently, time perception is subjective to the differences in one’s emotional state. There are various reasons for the disparate time perceptions in MDD and BD, which may be useful in distinguishing BD from MDD. However, most studies have only compared BD and healthy controls (HC) or MDD and HC [14, 19, 23, 30–32], whereas direct comparisons of time perception between BD and MDD have not been well investigated.

The previous studies, which tested durations of several seconds, used temporal production, tapping, and counting to investigate the temporal perception in patients with depression [24, 33]. Nonetheless, these studies did not adopt the methodological precaution to avoid the participants use a counting strategies. Thus, it is difficult to avoid the influence from the motor component, which has been considered as an important psychopathological feature of depression. Beyond that, previous findings have suggested that, compared with long-durations (>30s), the difference of temporal perception about short-durations (< 2s) between depression and healthy controls is smaller [23]. Thus, present study used a temporal discrimination task to which could exclude these influences caused by the motor response.

The time discrimination method requires subjects to distinguish between different lengths of time in a time estimation task. In this study, a psychophysical paradigm was used to measure the aberration of time estimation induced by MDD, BD, and HC, in real time during a temporal bisection task. The most representative method is the temporal bisection task proposed by Church and Deluty [34]. The experiment is simple and convenient, and has been used in many related studies on time perception. In this task, participants classify test visual stimuli as “short” or “long”, in comparison to a short and long duration “template” previously learned. This task includes four indicators: long response ratio, subjective BP, difference limen (DL), and Weber ratio (WR), which can simultaneously provide the accuracy of time judgment and time sensitivity [35]. Long response ratio refers to the proportion of participants who estimate the duration of a certain condition as close to the long duration, to the total frequency under that condition. BP is the point of subjective equality, in which the probe time duration corresponds to a 50% probability that the participant judges the interval as “long.” DL is the point estimated to be 75% of the long probability minus the point of 25% divided by 2. WR is the DL divided by the BP (for a more detailed, see [24, 34, 36]). Different BPs indicate that participants have different perceptions of the time interval. By comparing the BP between different test groups, it can be determined whether the subjective time interval of different groups is extended or shortened. The DL and WR are indicators of temporal sensitivity. The higher the WR, the lower the sensitivity to time. These results provide new insights into the role of time perception in the differential diagnosis of MDD and BD.

## Material and methods

### Patients

In this study, 30 patients diagnosed with MDD and 30 patients diagnosed with BD were recruited from Beijing Huilongguan Hospital and Zhumadian Psychiatric Hospital. 30 HCs were recruited from the Zhumadian city areas.

Inclusion criteria for MDD and BD patients included: a current major depressive episode; a primary diagnosis of MDD or BD according to the Diagnostic and Statistical Manual of Mental Disorders IV (DSM‐IV) criteria, by two experienced clinical psychiatrists; a score of more than 17 on the 17-item version of the Hamilton Depression Rating Scale (HAMD-17); normal or corrected-to-normal vision; Han Chinese ethnicity; and being right-handed.

Patients were excluded based on the following: other Axis I diagnoses, including anxiety disorders or schizophrenia; head injury resulting in loss of consciousness; electroconvulsive therapy in the preceding 6 months; uncontrolled hypertension, dyslipidemia or diabetes mellitus; a history of smoking or substance abuse/dependence; a history of heart disease. Female patients could not be pregnant, lactating, or taking oral contraceptives. All HCs met the same inclusion and exclusion criteria as those for MDD and BD patients, excluding the DSM-IV criteria for Axis-I psychiatric disorders.

The Ethical Committee of the Beijing Huilongguan Hospital reviewed and approved of this study (batch number 2016–72), which was conducted in accordance with the Declaration of Helsinki. All patients, or their parents and/or legal guardians for patients under 18 years of age, were given a detailed description of the study and they provided written, informed consent.

### Material

The experimental instrument was a desktop computer with a computer screen resolution of 1366 × 768 and a refresh rate of 60 Hz. The experimental program was compiled and implemented using E-prime2.0 software. The patients’ distance from the screen was approximately 60 cm, and all participants were tested in a quiet room.

The Hamilton Depression Rating Scale for depression-17, translated into Chinese in 1988, was used to assess the level of depression symptoms [37] (HAMD-17 score, >7 points and <17 points, mild depression; >17 points and <24 points, moderate depression; >24 points, severe depression) [38].

### Experimental procedures

The experimental process was divided into two phases: the training phase and the test phase. During the training phase, participants were required to learn two time intervals to strengthen their memory of long and short intervals. The time interval in the experiment was defined as the time between the appearance and disappearance of 2 × 2 black squares. The first phase was the training phase. For short duration training trials, "shorter time" in Chinese captions was displayed first in the center of the screen, followed by the appearance of a black square in the center of the screen, which disappeared after 400 milliseconds (ms). For long duration training, “longer time” in Chinese captions was displayed in the center of the screen, followed by the appearance of a black square in the middle of the screen, which disappeared after 1600 ms. Each type of trial was presented alternately, 5 times each.

**Fig 1:**
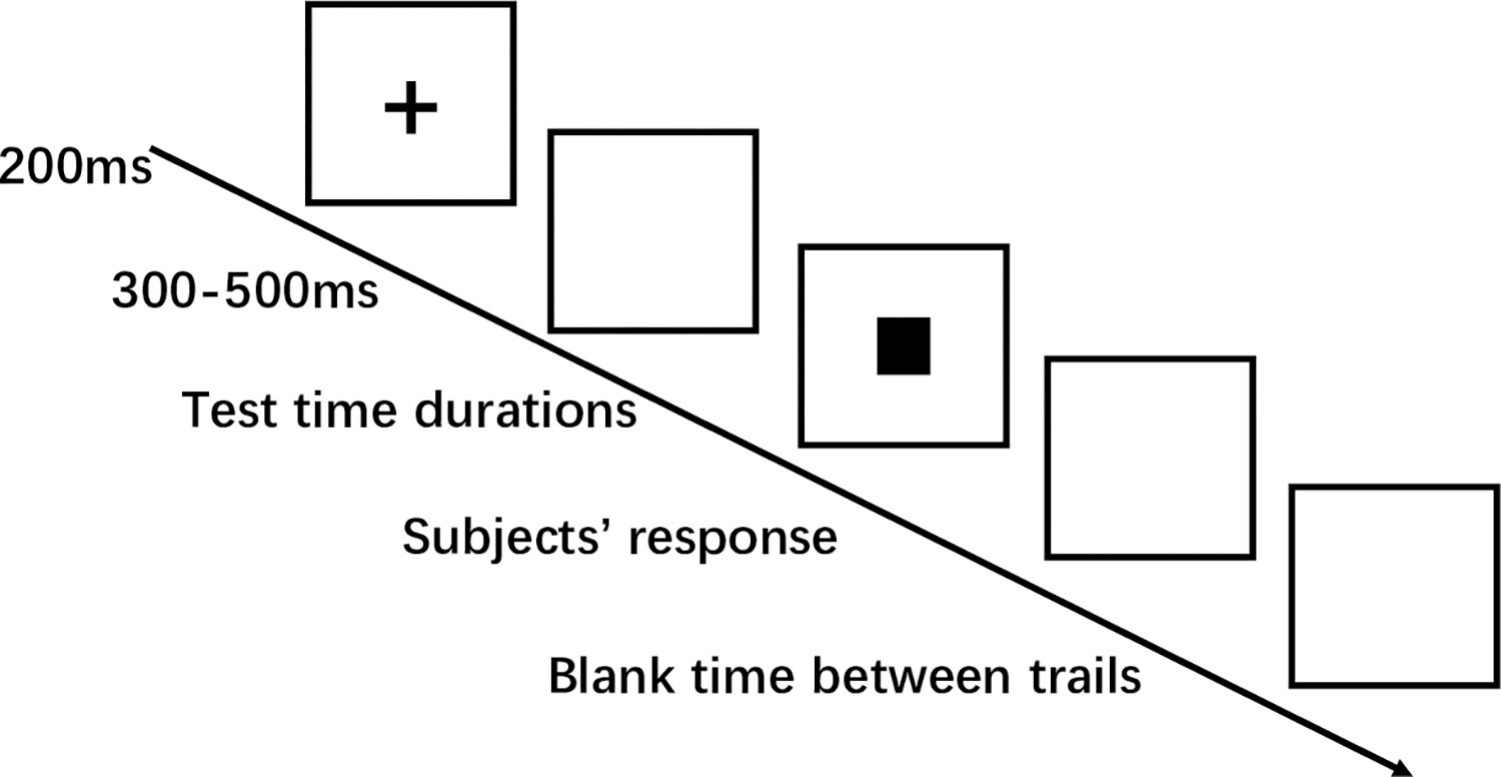
Paradigm of a trial in the test phase. In the test phases, participants were instructed to judge a series of time intervals as “short” or “long” based on the previously learned time interval.

The second phase was the test phase. The participants were instructed to make judgments on the length of a given time interval (rating intervals as "long" or "short") according to the previously learned understanding of time intervals. The specific procedure was as follows: a black cross (200 ms) appeared in the center of the screen. After the cross disappeared, the screen was be empty for intervals of 300–500 ms, followed by the appearance of a 2 × 2 cm black square in the center of the screen. Black squares were presented for 400, 600, 800, 1000, 1200, 1400, and 1600 ms, in random order. After the black square disappeared, the judgment interface appeared, and the participant was asked to press the "d" key to indicate a short interval or the "k" key to indicate a long interval. The blank time between trials was 1500–1800 ms. There were seven time intervals in the formal test phase: 400, 600, 800, 1000, 1200, 1400, and 1600 ms. Each time interval was presented 20 times, and the trials were completely random. A total of 140 trials were conducted. The experimental process is illustrated in Fig 1.

### Statistical analyses

After recording the data ratio of the participants’ judgment of “long” and “short” intervals, data were imported into SPSS 22.0 for corresponding statistical analyses. To verify the characteristics of the ratio of “long” judgment across the three groups of the formal experimental phase, the proportion of subjects making “long” judgments at each time interval was counted and analyzed [subject category (MDD, BD, and HC)× 7 (time intervals: 400, 600, 800, 1000, 1200, 1400, and 1600 ms)] and repeated three times for analysis of variance. Bonferroni analysis was used for post-hoc testing [Bonferroni-corrected *p* value for significance was *p* = 0.017 (0.05/3)]. To investigate the change in the BP across the three groups, a time point with a probability of 50% was calculated using the sigmoid curve in the curve expert software according to the proportion of long judgment (which is the subjective time BP) and the origin software was used to mapping. To explore the change characteristics of time sensitivity, the DL and WR of each subject was calculated. Subsequently, each indicator was used as the dependent variable, and groups were used as independent variables for analysis of variance.

## Results

### Demographic and clinical characteristics

There were no significant differences in general demographic characteristics (Table 1).

**Table 1:** General demographic profile of enrolled participants.

Items	MDD(n = 30)	BD(n = 30)	HC(n = 30)	X2/F/t	significance
**Gender (M/F)**	12/18	10/20	17/13	3.53	0.171
**Age**	27.07±9.98	26.09±10.27	27.13±4.66	0.01	0.994
**HAMD-17**	23.10±6.26	21.55±2.99		1.08	0.287

MDD, major depressive disorder; BD, bipolar disorder with depressive period; HC, group of healthy controls; HAMD, Hamilton Depression Rating Scale; Bold values, statistically significant at *p* < 0.05.

### Experimental result

#### 1. Long response ratio

At the end of this study, eight subjects were excluded because their data exceeding interval of ± three standard deviations (SD) from the mean. The data of 86 subjects entered the statistical analysis phase of the proportion of long response and response time, of which 30 were in the MDD, 26 in the BD, and 30 in the HC group. The results are presented in Table 2.

**Table 2:** Proportion of long responses across the three groups plotted in the bisection task (ms, $\bar{X}$ ± S).

group	400	600	800	1000	1200	1400	1600
**MDD (n = 30)**	0.09±0.12^&^	0.22±0.16^&^	0.48±0.22	0.71±0.16	0.81±0.16*	0.89±0.10	0.91±0.10
**BD (n = 26)**	0.08±0.15	0.18±0.16^#^	0.36±0.25	0.58±0.26	0.69±0.26	0.80±0.21^#^	0.85±0.20^#^
**HC (n = 30)**	0.01±0.03	0.08±0.10	0.33±0.25	0.59±0.22	0.82±0.13	0.91±0.10	0.96±0.07

MDD: major depressive disorder; BD: bipolar disorder with depressive period; HC: the group of health controls.

*BD vs. MDD, *p* < 0.017

^&^ MDD vs. HC, *p*<0.017

^#^ BD vs. HC *p<*0.017. *p*-value <0.05/3 = 0.017 (Bonferroni-corrected).

**Fig 2:**
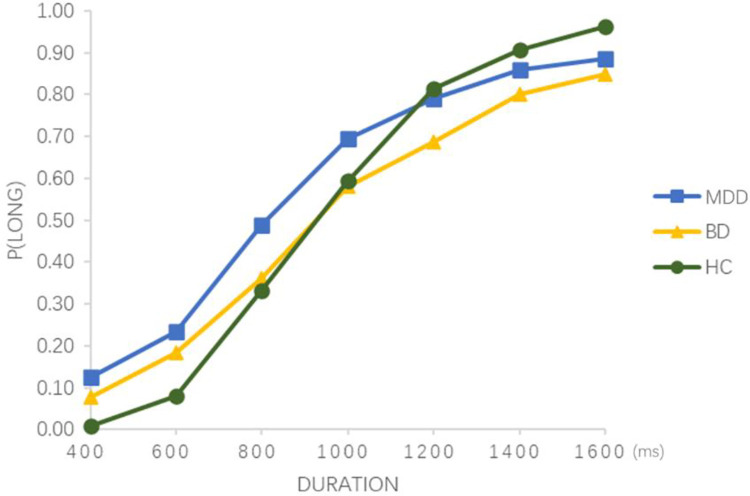
Proportion of long response in MDD group, BD group and HC group under seven durations. MDD, major depressive disorder; BD, bipolar disorder with depressive period; HC, healthy control group; P(LONG), proportion of long responses.

According to the judgment made by the subjects, a repeated measurement analysis of variance with 3 (subject type) × 7 (time interval) was performed on the long response ratio (Fig 2), with Bonferroni correction. The main effect of the groups was significant, *F*_(1, 83)_ = 3.622, *p* < 0.05, *η*^*2*^_*p*_ = 0.080. The main effect of the time interval was significant, *F*_(6, 498)_ = 547.468, *p*< 0.001, *η*^*2*^_*p*_ = 0.868, and the post-hoc test showed that the long responses ratio increased with the probe duration value. The long response ratio between any two durations was significantly different (*p*<0.017, Bonferroni-corrected). The interaction effect between subject type and duration was significant, *F*_(6, 498)_ = 4.656, *p* < 0.001, *η*^*2*^_*p*_ = 0.101. Further, simple effect analysis showed that at 400 ms, the long response of the MDD group was greater than that of the HC group (*p*<0.017, Bonferroni-corrected). There was no statistical difference between the MDD and BD groups, and the difference between the BD and HC groups was not significant. At 600 ms, the long response of the MDD and BD groups was greater than that of the HC group, and there was no statistical difference between the MDD and BD groups. There were no significant differences in the long response of the MDD, BD, and HC groups at 800 ms (*p*>0.017, Bonferroni-corrected). At 1200 ms, the long response of the MDD group was greater than the BD group (*p*<0.017, Bonferroni-corrected), and the long response of the BD group was smaller than the HC group. There was no statistical difference between the MDD group and the HC group. At 1400 and 1600 ms, the long response of the BD group was smaller than that of the HC group (*p*<0.017, Bonferroni-corrected). There was no statistical difference between the MDD and BD groups, and the difference between the BD and HC groups was not significant (Fig 2).

#### 2. The subjective bisection point (BP), difference limen (DL) and Weber ratio (WR)

As the data of two subjects in the MDD group and four subjects in the BD group did not follow the characteristics of the sigma equation, their fitting parameters exceeded the three standard deviations of the fitting parameters of other subjects. These patient data were deleted when we calculated the BP, DL, and Weber ratios. A total of 82 participants’ data were used to calculate the BP value, the value of DL, and the WR value, 30 in the MDD group, 22 in the BD group, and 30 in the HC group.

**Fig 3:**
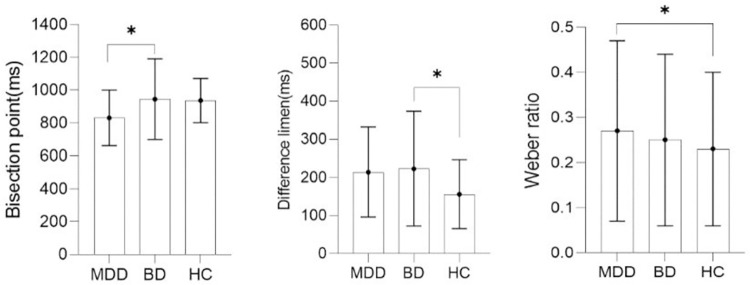
The BP, the DL and WR for three groups. MDD: the group of major depressive disorder; BD: the group of bipolar disorder with depressive period; HC: the group of health controls; BP: the subjective bisection point; DL: difference limen; WR: Weber ratio; * *p*< 0.05.

The long response ratio to the sigmoid curve in the Origin software was used to calculate the value of BP, the value of DL, and the value of WR. The one-way ANOVA followed by post hoc tests (LSD) revealed significant differences among the HC, MDD, and BD groups in the BP values and WR values, *F*_(2, 81)_ = 3.462, *p* = 0.036 vs. *F*_(2, 81)_ = 3.311, *p* = 0.042. The difference in the value of the DL among the three groups was not significant, *F*_(2, 81)_ = 2.597, *p* = 0.081. Post-hoc tests showed that the value of BP in the MDD group was less than that in the BD group (*p* = 0.027), and the value of BP in the MDD group was less than that in the HC group (*p* = 0.027). Similarly, post-hoc tests showed that the WR values in the MDD group were larger than those in the HC group (*p* = 0.022). Details are presented in Fig 3.

#### 3. Relations between BP, DL and WR with depression symptom severity in BD and MDD were examined by Pearson correlations

The Pearson correlation analysis showed that no significant relationships were observed among BP, DL, WR, and *HAMD*-*17* scores in the MDD and BD groups, respectively (Table 3).

**Table 3:** Relations between BP, DL and WR with depression symptom severity in BD and MDD.

group	item	*r*	*P*
**MDD(n = 30)**	BP	-0.34	0.860
	DL	0.24	0.198
	WR	0.25	0.180
**BD(n = 22)**	BP	0.15	0.506
	DL	-0.34	0.122
	WR	-0.392	0.071

MDD: the group of major depressive disorder; BD: the group of bipolar disorder with depressive period; BP: the subjective bisection point; DL: difference limen; WR: Weber ratio.

## Discussion

This study examined the time perception characteristics of MDD, BD, and HC groups in the range of 400–1600 ms and found that compared with healthy controls, there was a significant difference in time perception between the MDD and BD groups. The value of BP in the MDD group was significantly smaller than the value of BP in the BD and HC groups, which indicates that the subjective judgment of the MDD group on the time interval exhibits “extended” phenomenon compared with the BD and HC groups. Similarly, the MDD group had a larger WR value than the HC group, which suggests that MDD patients are less sensitive to time than non-disordered individuals.

In the range of 400–1600 ms, the long response ratio of all subjects increased with the increase in time interval, which means that the MDD, BD, and HC groups can make a relatively accurate estimation of the time interval in this duration. However, compared with healthy controls, the MDD group overestimated these short time intervals (400ms and 600ms). Although, recent results not show a statistically significant difference of the proportion of long responses for the longer stimulus duration (1200, 1400 and 1600ms) between the MDD group and HCs. But, from Fig 2, there was a trend that MDD group underestimate these longer stimulus durations compared with HCs. This indicates that MDD patients showed variations in the processing of temporal information. Research by Dan Tao et al. [39] showed that compared with control groups, both clinical and subthreshold depression groups tended to overestimate short durations and underestimate long durations, while there was no significant difference in the value of BP between either the subthreshold depression group or the clinical depression group, and the control group. One possible reason for this difference is that the inclusion criteria of participants in the present study were different from those of the studies by Tao et al. The subjects in the studies by Tao et al. were divided into two groups: patients with clinical depression and a control group. The depression evaluation of patients with depression was based on the “Diagnostic and Statistical Manual of Chinese Mental Disorders, 2nd Revision (CCMD-2-R).” The inclusion criteria for major depressive disorder patients in the present study was based on the DSM-V. Moreover, the patients in this study scored more than 17 on the 17-item version of the Hamilton Depression Rating Scale (HDRS), yet they were not grouped further based on the HDRS scores. Rather, subjects were divided into three groups: MDD, BD, and HC groups. A meta-analysis on time perception [23] suggested that due to inconsistent classification criteria in the time perception task, different individuals may be divided into different groups which would result in differences in the final analysis. The results reflected in long time interval response and the lower value of BP in the MDD group, compared with the HC group, illustrated that patients with major depressive disorder feel time as extended in certain conditions, which is similar to the results of mounting studies such as Wyrick RA [22], Kitamura T [40], and Kornbrot DE et al. [18].

This present study found that compared with HCs, the BD group showed overestimating shorter time intervals and underestimating longer time intervals (i.e., BD group overestimates 400ms, 600ms time intervals and underestimates 1200ms, 1400ms, 1600ms time intervals). This indicates that patients with bipolar disorder in the depressive period have shown variations in the processing of temporal information. A study on timing variability in schizophrenia and bipolar disorder [32] by Bolbecker AR illustrated that timing variability was significantly higher in non-psychotic bipolar disorder or bipolar disorder with psychotic features groups, than in healthy controls. In the present study, the time perception of BD patients was similar to that of clinical depression groups in the study by Tao et al. [39], which showed that BD patients in the depressive period may follow the change in time perception trend of the depressive group. Tao’s study pointed that work memory play an important role in the time perception. Meanwhile, some studies showed that impaired working memory is common in individuals with bipolar disorder which may cause these variations in the processing of temporal information [41, 42]. However, the BP values in Tao’s study were not significantly different between patients and healthy controls. This was likely due to the insufficient number of BD patients or the fact that these patients were not very sensitive to the time intervals.

It should be noted that the value of BP in the MDD group was lower than that of the BD group in the present study, which means that compared with BD patients, MDD patients likely perceived time as extended. These results indicate that the group with major depressive disorder probably has different time perception than the group with bipolar disorder in the depressive period.

The scalar expectation theory (SET) [43] is the main theory explaining intervals and time perception, which has been extensively confirmed [44–46]. The SET divides the process of time perception into three stages: internal clock, memory, and decision. According to SET, a pacemaker generates pulses at a certain frequency. There is an attention switch between the pacemaker and the accumulator, through which the pulses are collected into the accumulator and stored in the reference memory. Then, as a decision-making process, the current duration is compared with the sample duration to make a time judgment. An increasing number of studies have suggested that variations in time perception are influenced by emotions and attention [13, 47, 48]. The change in emotions will influence attention, which would control the switch. The results of event-related potential (ERP) in the study by Tao et al. [39] showed that the ability of time processing was reduced in depression patients. As described above, attentional deficits and emotional processing changes in patients with MDD and BD in the depressive period may influence their time perception.

Previous studies indicated that the dopamine system may be related to the internal clock [45, 49]. The one known study about Parkinsons disease patients found that these patients exhibit time-distance perception impairment due to dopamine degeneration projected into the basal ganglia [50]. Basal ganglia have been found to be involved in the secretion of dopamine in the brain, and it has been confirmed that basal ganglia are involved in the development of many mental diseases [51, 52]. In the study of time perception, it was found that the basal ganglia are a specific time information processing system [43]. From the perspective of dopamine secretion, it has been found that reduced dopamine secretion can distort time perception [53]. The volume of the basal ganglia and hippocampus is reduced in both MDD and BD patients, but patients with major depressive disorder are the worst affected in this regard [54]. Overall, it is likely that there is a difference in the time perception of patients with major and bipolar disorder, due to the occurrence of lesions in the brain area related to time perception.

This present study did not find any correlation between BP, threshold of difference, WR, and severity of depression in patients with depression, which is consistent with previous research. In other words, there may be no relationship between the change in time perception in patients with depression and the severity of depression [55]. Therefore, the change in time perception of patients with depression may be a quality change that may not be related to the severity of the disease. However, the results of this study are different from those of Dan Tao et al. Their study found that there is a correlation between the changes in time perception in patients with depression and the severity of depression. The reason for this difference may be related to the difference in the two research subjects, as Tao’s research subjects included subjects with subliminal depression [39].

In conclusion, the present study found that compared with healthy controls, the subjective time and time sensitivity of MDD patients and BD patients in the depressive period changed to some extent. Compared with the BD depressive patients, the major depressive disorder patients likely felt time extended. The group with major depressive disorder had different time perception than the group with bipolar disorder in the depressive period, which may be used as a biomarker to distinguish these two disorders.

The present study has some limitations. First, the number of participants in this study was small. Second, the present study recruited patients who had scores of more than 17 on the 17-item version of the Hamilton Depression Rating Scale, but did not distinguish these patients according to their scores. Furthermore, the underlying cognitive and neurochemical mechanisms of time perception in patients with MDD and BD in the depressive period needs to be studied.
